# The Future Farm project in Western Australia: testing multidisciplinary options for a sheep-crop farming system in a Mediterranean region

**DOI:** 10.1093/af/vfag013

**Published:** 2026-04-07

**Authors:** Graeme B Martin, S Senthil Murugan

**Affiliations:** UWA Institute of Agriculture and UWA School of Agriculture and Environment, The University of Western Australia, Crawley 6009, Australia; Livestock Research Station, Kerala Veterinary and Animal Sciences University, Thiruvazhamkunnu, Palakkad District, Kerala 678601, India

**Keywords:** alternative forages, animal welfare, conservation and biodiversity in farming, livestock-crop farming systems, methane emissions

## Abstract

**Graphical Abstract:**
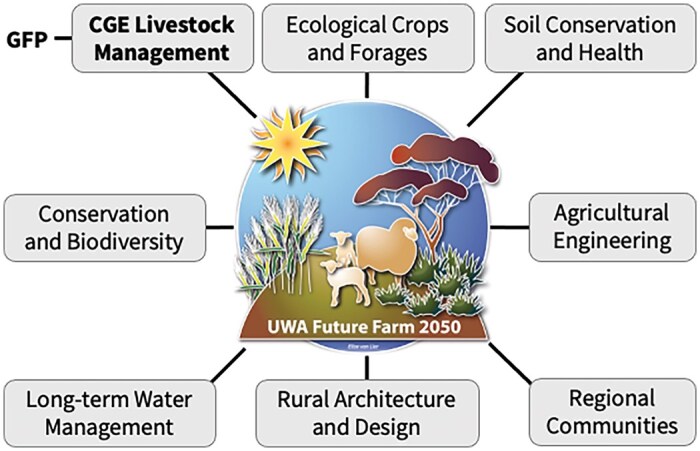
A full-scale, commercial model farm that offers opportunities for evaluation and demonstration of innovative practices that integrate the CGE management of Merino sheep with cropping, and the management of natural resources, the environment, and biodiversity. International relevance is ensured by connection to the *Global Farm Platform* (GFP). Created by E van Lier and GB Martin.

A full-scale, commercial model farm that offers opportunities for evaluation and demonstration of innovative practices that integrate the CGE management of Merino sheep with cropping, and the management of natural resources, the environment, and biodiversity. International relevance is ensured by connection to the *Global Farm Platform* (GFP). Created by E van Lier and GB Martin.

Implications

- The challenges for livestock farming are multi-disciplinary so solutions are best developed and demonstrated on real-world farms because farming enterprises are multidisciplinary.
- Our model farm project is based on a holistic assessment of the “critical zone” that sustains terrestrial life, and supports “clean, green and ethical (CGE) livestock production” and “conservation cropping,” while meeting the needs of farming communities.
- Our CGE livestock system uses sheep breeding and novel forages to address issues such as methane emissions and animal welfare, and thus increase productivity, while seeking solutions for the management of biodiversity and landscape.

## Introduction

**Figure 1.**
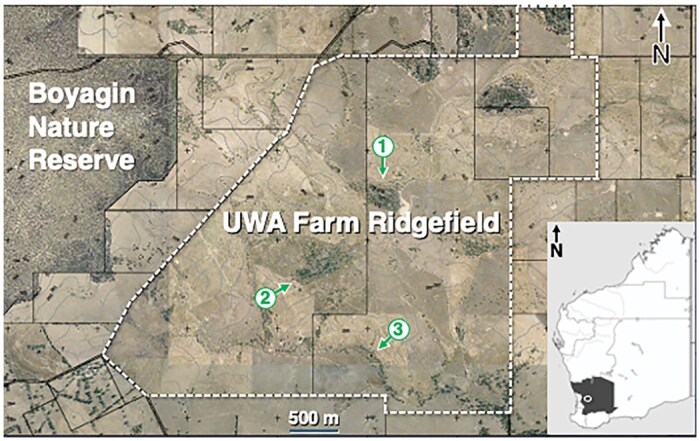
An aerial photograph of *Ridgefield* with the eastern part of the adjacent Boyagin Nature Reserve (6,700 ha). The small remaining areas of native bushland are evident, as is the small river running west-east inside the lower boundary. The photograph was taken in autumn. Green arrows indicate bushland remnants (1, 2) and a creek line (3). Inset: map showing the regions of Western Australia, with the shaded area indicating the “grain belt,” and the circle indicating the location of *Ridgefield*.

Farmers of the future will need to continue producing food for humanity, but, as custodians of much of the planet, they will also need to seek solutions for the conservation of landscape and biodiversity. Farming is already multidisciplinary, but this broader remit will add extra dimensions to their enterprises, and solutions will need to be sought from a wider variety of sources. Future farmers will need to connect with research organizations, rural communities, industries, local authorities, and, most importantly, educators. Given the complexity of these interactions, particularly for grazing livestock, holistic solutions can be best evaluated and demonstrated on real-world farms. In 2009, this situation led The University of Western Australia (UWA) to establish *UWA Future Farm 2050*, recently renamed *UWA Best Practice Farming Systems Project*  https://www.uwa.edu.au/institutes/institute-of-agriculture/UWA-Farm-Ridgefield. The project is located on *Ridgefield*, a 1588 ha property (S 32°30′23″; E 116°59′31″) in the “grain belt” of Western Australia (Figure 1). The climate is Mediterranean, and the average annual rainfall is about 445 mm, most of which falls in the cooler months (April to September).

From the beginning, it was important that *Ridgefield* not be seen as a classical research station covered by small trials, but as an agricultural business that would be a source of inspiration for the future of sheep-crop enterprises. Income is generated by crops (cereals, canola) and by Merino sheep (meat, wool), with the normal obligatory commitments to water and soil management. However, in contrast to most agriculture projects, *Ridgefield* also embraces biodiversity conservation, the human environment, and community development. The typical agricultural disciplines are therefore complimented by various others, including architecture, landscape architecture, engineering, hydrology, geophysics, rural development, and health science. In addition, the project aims to strengthen links between food producers and food consumers by engaging with industry, local authorities, and schools.

In 2012, it became clear that many of these goals resonated with those of a cognate project in the United Kingdom, the *North Wyke Farm Platform*, so forces were joined to initiate the Global Farm Platform (GFP) with the help of funding from the Worldwide Universities Network. An important initial outcome was a position statement for the future of livestock industries (Eisler et al., 2014). Another early member of the network, Silent Valley Farm (*Thiruvazhamkunnu*) in southern India, is described in a companion paper (Murugan et al., 2026). The basic questions are the same for both model farms, but the solutions differ because of the major differences in geographical and socio-economic context.

## The Natural Resources—Assessment and Management

Quantification of land resources is essential for decision-making and long-term planning, so *Ridgefield* has its own GIS transmitter and has been mapped to 2 cm x 2 cm squares, with matching to the landscape and the surrounding areas, including the neighboring state conservation park (Figure 1).

### Soil assessment and management

As a foundation for decisions for land use, a complete survey and analysis of the soils was begun immediately after the farm was acquired and it continues, often in the context of practical classes for science degrees. The database includes classical soil descriptions, digital soil mapping, publicly available web-GIS sources, hydrological data, and shallow geophysical methods (e.g., electrical conductivity, gamma radiometric mapping). This information is integral to a holistic understanding of interactions among the atmosphere, hydrosphere, lithosphere, and biosphere—the entire space from top of tree canopy down to aquifer—known as the “critical zone” (CZ). Within the CZ, energy is transported and transformed, and various weathering and soil formation processes enrich, leach, or transform organic and inorganic components. In other words, CZ science studies the zone that sustains most terrestrial life. *Ridgefield* is connected into the international *Critical Zone Exploration Network* (CZEN, https://www.czen.org), as the first CZ Observatory (CZO) in the southern hemisphere (Leopold et al., 2024).

### Land resource allocation

The combination of data from the CZO, hydrology, geography, and crop yields allows us to plan the subdivision of the farm according to enterprise, and then progressively modify the plan as we gather new data. Land is allocated on the basis of suitability for cropping (continuous or rotational), grazing (continuous or rotational), permanent native vegetation reserves (water courses, biodiversity corridors, erosion prevention, and land otherwise unsuitable for productive agriculture), and infrastructure projects (buildings, stock handling, communications, roads, fences, water capture). To date, about 380 ha is allocated to permanent pasture or ecosystem management, 570 ha to pasture-crop rotation, and 570 ha to grain crops.

## Commercial Enterprise 1: “Clean, Green and Ethical” Sheep Production

*Ridgefield* has a single livestock enterprise—a flock of around 4,000 female Merino sheep. This genotype has dominated the Australian sheep industry for more than 200 years and is particularly well adapted to the Mediterranean and semi-arid regions of the continent. Until the 1990s, fine wool was the dominant commodity, but market pressures have seen the Merino become dual-purpose, also producing meat for local and export markets. Globally, the value of animal-sourced food is being questioned but, to feed humanity, all options must be considered, and grazing ruminants offer the massive advantage of converting biomass that humans cannot digest into meat and milk, as well as adding value to farmland not used for crops (Eisler et al., 2014; Martin, 2024). Nevertheless, the Australian Merino industry needs to respond to the major societal pressures that effectively translate into market pressures, especially in markets where there is discretionary spending power—the local and export markets for Australia.

We responded to this situation during the early part of the 21st century by developing and promoting a vision for “clean, green and ethical” (CGE) livestock management (updated Martin, 2022). “Clean” involves minimizing the use of drugs, chemicals, and hormones; “green” involves minimizing the impact on the environment (particularly, the greenhouse gas, methane); and “ethical” focuses on animal welfare. For the *Ridgefield* flock, the two most important issues are: i) methane emissions—the science of ruminant methane emissions has advanced considerably since the alarm was raised by *Livestock’s Long Shadow* (Steinfeld et al., 2006); for example, we now know that blocking methane synthesis in the rumen can improve animal efficiency because carbon that would have escaped is redirected into production, so reducing methane emissions is a “win-win” situation (review: Martin, 2024); ii) animal welfare, where progress is being improved by technological developments that effectively allow managers to know when a sheep is stressed (Maloney et al., 2013), how sheep can cope with stress (Babington et al., 2025), and discovery of the genes that control susceptibility to stress (Ding et al., 2024).

**Figure 2.**
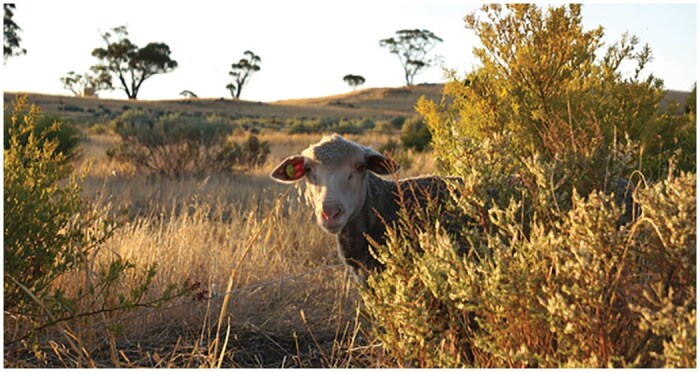
A Merino ewe in native Australian shrubs showing the degree of shelter. This photograph was taken in autumn, after the senescence of the normal annual pastures, when the shrubs act as “edible shelter” and provide sufficient green feed to maintain body weight—an important consideration because autumn is the normal breeding season (Masters et al., 2023).

In both of these areas, global knowledge and perspectives are evolving. There is now a strong argument that we have been over-estimating the Global Warming Potential of ruminant methane (see Martin, 2024); animal welfare has seen the development of holistic perspectives in *One Health* and *One Welfare* (Pinillos et al., 2016), and welfare now addresses interactions among health, nutrition, and management, with a general drive to minimize stress from all sources (review: Martin, 2024). For the management and development of the *Ridgefield* flock, we are responding on two fronts:

1. Breeding—our two general goals are: first, use the power of genetics to optimize the sheep flock for the needs of CGE management, targeting traits where the heritability is known, or where we have evidence of genetic variation; second, develop a robust “easy-care” genotype suited to extensive management (review: Martin, 2022). For this purpose, the Merino provides a strong foundation as a low-cost, high-profit genotype that is adapted to our environment, thus providing a foundation that we can build on:
  - Fecundity and rearing success are heritable, and the ideal target is for every ewe to wean twins;
  - Residual feed intake is also heritable so can be targeted to increase feed efficiency;
  - Disease resistance: helminth infection, compounded by resistance to medication, is a major issue; it directly reduces productivity, is largely responsible for flystrike (myiasis) and therefore the ethically controversial practice of “mulesing” (Colvin et al., 2022); resistance to helminth infection and to flystrike are both highly heritable (Greeff et al., 2014);
  - Methane production, now known to be heritable, can be reduced (Aldridge et al., 2025);
  - Calm temperament—in addition to reducing responses to stressors and thus improving health, this trait is associated with reproductive capacity, growth, meat quality, and milk production (Ding et al., 2024; Kitagawa et al., 2024);
  - A short tail—in the long term, this might be useful to avoid the ethical issue of tail-docking (Hodge et al., 2025).
2. Alternative grazing systems meshes with the whole-farm, holistic nature of the *Ridgefield* project; we have begun using Australian indigenous plant species, particularly the perennial shrubs, because they: i) are adapted to our climate and soils, and are deep-rooted (drought-resistant); ii) lower the level of saline ground water; iii) increase soil carbon content; iv) reduce wind speed thus mitigating soil erosion and evaporation of soil moisture; v) provide shelter for small plants. Importantly, in contrast to imported pasture species, they are indigenous and thus provide a reservoir for beneficial invertebrates and thus for maintaining biodiversity. The shrubs can be permanently established in areas where cropping is not feasible and they can be mixed in with traditional fodder grasses and legumes (Revell et al., 2013).
  For grazing sheep, the benefits include: i) provision of good biomass, particularly in autumn (Revell et al., 2013); ii) reduction of methane emissions (Durmic et al., 2022); iii) combatting gastrointestinal nematodes (Kotze et al., 2009); iv) prevention of rumen acidosis (Hutton et al., 2012); v) provision of “edible shelter” (Figure 2) that improves neo-natal lamb survival, reflecting the general importance of shade and shelter for the future of grazing livestock systems (review: Masters et al., 2023).

For the shrub innovation, Dean K. Revell and Philip E. Vercoe were awarded an *Australian Museum Eureka Prize* in 2013. In 2015, the *Ridgefield* project was accepted into the FAO *Repository of best-practice models in grassland, rangeland, and pastoral management*. Recently, as evidenced in this Special Issue of *Animal Frontiers*, it also contributed to the Technical Recognition of the *GFP* at the 2025 FAO Global Conference on Sustainable Livestock Transformation (https://www.fao.org/one-health/highlights/recognition-of-good-practices-and-innovations).

## Commercial Enterprise 2: Conservation Cropping

*Ridgefield* is situated in the “grain belt” of south-western Australia (Figure 1), so wheat and canola are major contributors to farm income. We use “no till” cropping, a practice that has been extensively adopted by dry-land grain growers in our region because of a variety of benefits: timeliness of seeding in relation to autumn rainfall events; use of crop residues to reduce the risk of soil erosion after the harvest; improved retention of soil moisture as seeding time approaches; maintenance of soil structure; and increased soil microbial function (Cookson et al., 2008; D’Emden et al., 2008; Ward et al., 2012). The issue of soil moisture is becoming more important as climate change leads to less rainfall in a more unpredictable pattern. At present, no-till practices rely on herbicides for weed control, but herbicide resistance has been accelerated by complex interactions among genetics, weed management practices, and weed biology. To drive down and sustainably maintain low weed populations, we must combine best-practice use of herbicides, control weed seeds at harvest (Norsworthy et al., 2012; Walsh et al., 2013), and use state-of-the-art technology that avoids whole-of-paddock tillage with a robot that targets individual plants (Walsh et al., 2020). A similar problem is arising with resistance of insect pests to insecticides, so there is developing interest in holistic alternative approaches, as embodied in the concepts of integrated weed management and integrated pest management (Llewellyn et al., 2007; Norsworthy et al., 2012; Macfadyen et al., 2014). We are exploring the potential of remote sensing to map soils, crops, and areas with pest infestations, so that applications of fertilizers, chemicals, and mechanical weeding can be targeted.

On *Ridgefield*, we are confronting these challenges, but we also need to deal with competition within the farm for land resources by other enterprises, particularly livestock and ecosystem restoration (Fisher et al., 2012).

## Management of the Environment and Biodiversity

As landowners and managers, farmers and graziers are responsible for about half of Australia’s landscape, so they must play a major role in the management of the environment and the conservation of biodiversity.

### Ecosystem restoration

On *Ridgefield*, there is significant potential for improving the conservation of biodiversity assets because the woodland remnants represent some of the historical vegetation cover that existed before extensive clearing for agricultural development (Figure 1). Most of these remnants are located on lateritic breakaways that are not suitable for agriculture. First, grazing sheep were excluded from the remnants; second, native vegetation was re-established between the remnants to improve connectivity among them, and alongside water courses to initiate the restoration of riparian ecosystems (Figure 1). This work also benefits agricultural production through ecosystem services (Kremen and Miles, 2012; Standish and Hulvey, 2014; Kragt and Robertson, 2014).

### “Carbon farming” opportunities

The need to reduce carbon emissions and improve carbon sequestration led to the concept of “carbon farming” that, in principle, would allow farmers and land managers to earn “carbon credits” that could then be sold as a part of the farm business. The establishment of native shrubs for grazing, outlined above, could fall under this scheme. More aspirational is planting of native trees at paddock scale (but not as plantations for harvest) to contribute toward biodiversity conservation and landscape health, as well as livestock shade (mentioned above), and ecosystem services for agriculture (Bradshaw et al., 2013; Lin et al., 2013). Evidence is emerging that planting multiple species will sequester at least as much carbon as planting only the single most productive species (Hulvey et al., 2013; Perring et al., 2015). The *Ridgefield* project offers an opportunity to test these complex hypotheses through long-term studies, so it joined *TreeDivNet*, the global network of research on biodiversity and ecosystem functioning of woody ecosystems (www.treedivnet.ugent.be; Perring et al., 2012).

## Agriculture Needs People

Like most universities, UWA is home to many disciplines other than those that are focused on agriculture and can therefore offer input into the wide variety of issues that confront farm management, farming life and the welfare of the local community.

### Water management

Like much of our region, there is limited access to potable water for livestock, and there is a risk that long-term decisions about, say breeding, could be disrupted by a single poor year of rainfall, wasting many years of progress. For *Ridgefield*, we analyzed rainfall runoff and then constructed a dam of 5,500 m^3^ that would fail to fill in only 2 years out of 50. This would be sufficient to provide water for 1,500 sheep plus extra for spraying and fire-fighting.

### Housing for people—on the farm and in the towns

Transport of materials, skills, and labor for conventional building projects is very costly, so regional residents are usually forced to build poorly designed and constructed homes that are ill-suited to the climate. For *Ridgefield*, Professor Patrick Beale (School of Architecture, Landscape and Visual Arts, University of WA) built a prototype house for the farm manager, using state-of-the-art design and innovative, low-cost construction processes based on “flat-pack modules” that are factory-built to high standards, incorporate environmentally efficient features, and can be assembled quickly. Roof insulation exceeds the highest rated category for domestic insulation and the eaves extend 1.2 m beyond the perimeter, keeping summer sun off the external walls while admitting as much winter sun as possible. Rooms are equipped with opposing sets of louvres to control airflow and permit rapid cooling at night. The structural frame and floors are built from renewable hardwood and timber certified by the *Forest Stewardship Council* (https://fsc.org/en). The roof cladding is coated with ceramic nanoparticles and reflects up to 40% more heat than conventional corrugated sheets. Windows are “low-E glass,” and the external wall cladding is a fire-resistant, low-density, fibre cement sheet. The floor deck rests on stilts that assist summer cooling, reduce the risk of termite infestation, and mitigate the effects of seismic activity (the region experiences about 100 small tremors per year). Self-sufficiency in water is delivered by collecting rain from the roof into two 128 kL tanks. All electricity is provided by photovoltaic panels that generate 10 kWh and are combined with state-of-the-art batteries and monitoring.

### Community relationships

One of themes of the *Ridgefield* project is “contented farmers living in vibrant communities,” so it was important that the university be seen as a valued member of the local community, based around the town of Pingelly. The project has thus become a unique and interesting space where academics and students from the arts and sciences sit side-by-side and work alongside the local community in a shared vision.

1. Local economy—as far as possible, farm purchases are made locally, and farm staff contribute to local community activities; visits by students, international delegations, research teams, and artists-in-residence help to bring life and vitality to the town; at “open days,” held every 2 years, the local community is invited to inspect the project; links have been made with the local schools, and presentations have been given to the local shires.
2. Infrastructure—in addition to the farmhouse described above, Professor Beale designed and helped build the *Pingelly Recreation and Cultural Centre* (https://www.pracc.net.au), a winner of architectural awards at local, national and international levels.
3. Modern-day farmers are confronted with complex social and ecological challenges, for which they have no experience or training, and threaten their mental well-being. Dr Susan Bailey (now at Edith Cowan University) developed the practice of “eco-social work,” bringing together social and ecological domains, as a way to promote interdisciplinary collaborations to benefit urban and rural communities, non-human life, and the land. She also forged a relationship with the local indigenous population in recognition of the fact that *Ridgefield* is located in a region in south-west Western Australia, *Gnaala Karla Boodja*, where farmers and legislatures have influenced the landscape for more than 200 years since colonial settlement. An important outcome of this relationship was an *Acknowledgement of Country* on a sign at the major entrances to the farm: “The University of Western Australia acknowledges that Ridgefield is situated on *Gnaala Karla Boodja*, and that Noongar people are the spiritual and cultural custodians of the land, and continue to practise their values, languages, beliefs and knowledge.”
4. Aging population—Pingelly, like many towns in regional Australia, is struggling with an exodus of young people and is poorly equipped, materially and intellectually, for the consequences; retirees move to the city, away from family and life-long friends. Dr Loretta Baldassar (now at Edith Cowan University) is directly addressing this issue with her project, *Aging in Place*, launched in 2021 (Baldassar et al., 2021).

### The next generation of agricultural professionals

The *Ridgefield* project has a 15-year history in education at all levels and is able to provide both local context and a worldwide perspective to course development.

1. Secondary education—Australia exports about 60% of its agricultural produce. Despite this importance in the national economy, the demand for agricultural professional people is 3-fold greater than supply, so there is pressure to improve the flow of students into university degree programs (Graham et al., 2025). The *Ridgefield* project informed the development of two new secondary school courses that were launched in 2024: i) Agribusiness, to attract students interested in commerce; and ii) Agricultural Science and Technology, to attract students interested in science and engineering. This work involved a partnership (UWA, Murdoch University, Edith Cowan University, Curtin University) operating under the auspices of the WA government’s Secondary Curriculum and Standards Authority.
2. Worldwide—*Discover Best-Practice Farming for a Sustainable 2050—*this Massive Online Open Course (MOOC) is based on *UWA Future Farm 2050* and challenges learners to consider the implementation of future farming concepts in their own part of the world. It was launched by Coursera in 2017 and, by January 2026, had accumulated 45,600 total learners. https://www.coursera.org/learn/best-practice-farming-sustainable-2050

## Conclusions

As a full-scale, commercial model farm, the *Ridgefield* project encourages staff and students in a wide variety of university-based disciplines to engage with agricultural industries and rural communities. The farm offers opportunities for evaluation and demonstration of innovative practices that integrate the CGE management of Merino sheep with cropping and the management of natural resources, the environment, and biodiversity. In contrast to most farming projects, the *Ridgefield* project also recognizes the importance of people in meeting the challenges facing agriculture so embraces engagement with the local community by providing input into infrastructure and the resolution of community issues. International relevance is ensured by connection to three global research networks: the *GFP*, the *CZEN*, and *TreeDivNet*. There is real scope for extending the *Ridgefield* concept to a variety of geographical and socio-economic environments, and thus greatly improve our ability to “feed the world without destroying the planet.”

## Author Biographies

**Graeme B. Martin** gained a PhD from The University of Western Australia (UWA) and then worked in France (INRAe) and in Scotland (MRC) before returning to UWA, where he subsequently reached the level of Professor (Chair) and is now Professor Emeritus. His research focused on how environmental factors affect reproduction, mostly in the sheep and the emu, using techniques in neuroscience, endocrinology, metabolism, behavior, reproductive technology, molecular biology, and computer modeling. From this experience, he developed “clean, green and ethical” livestock management, a theme he then incorporated into “UWA Future Farm 2050.” He is a Lifetime Member of the *Society for Reproductive Biology* (Australasia) and a Fellow of the *Australian Association of Animal Sciences*. In 2019, he was elected as an Associate Member of the *Academie d’Agriculture de France* and, in 2021, was awarded the 2021 Marshall Medal by the *Society for Reproduction and Fertility* (UK).

**S. Senthil Murugan** earned his MVSc from Tamil Nadu Veterinary and Animal Sciences University, Tamil Nadu, and his PhD from Gandhi Gram Rural Institute (GRI; Deemed University), India. He began his professional career as an animal nutritionist in the cattle feed industry and worked in various units of the Kerala Veterinary and Animal Sciences University (KVASU). He is currently serving as Unit Head, using his experience to implement a best-practice dairy farming system at *The Silent Valley Farm*, Livestock Research Station, Thiruvazhamkunnu, KVASU. His primary research focus includes enhancing the quality of lingo-cellulosic fibrous materials with the aim of mitigating enteric methane emissions, and developing an area-specific mineral mixture to promote sustainable livestock production. His collaborative research with the Board of Research in Nuclear Sciences (BRNS), Bhabha Atomic Research Centre (BARC), Mumbai, Government of India, to evaluate fibrolytic enzymes through *in vitro* digestibility studies, has been widely adopted in total ration formulations by dairy farmers. He has developed encapsulated nutrients (probiotics, nano minerals, amino acids) as feed additives for ruminants, at laboratory level. He has been honored with a Gold Medal and recognized with the ICAR Best Teacher Award.
